# Reflecting on the importance of theory-informed qualitative research in people with chronic respiratory disease and their carers

**DOI:** 10.1177/14799731231185128

**Published:** 2023-06-21

**Authors:** Emma L Giles, Samantha L Harrison

**Affiliations:** 1 School of Health and Life Sciences, 5462 Teesside University, Middlesbrough, UK

Theory can be defined as “a formal logical explanation of some events that includes predictions of how things relate to one another.”^1^ Theory can be used in research in many ways. It can be used to help craft, develop, and guide research questions. It can help decide what data you want to capture. It aids in the interpretation and analysis of findings, and it can help to explain the phenomena of interest.^2^ Often, theories pertain to how people act and behave in certain ways, how a society works, and how businesses and organisations run. Indeed, there are multiple theories which can generate confusion and uncertainty in how to apply theory to research. The consideration of theory is important, particularly at an early stage in research.^3^ This is to help give research a clear direction. Whilst theory can be applied both a priori and retrospectively, it is the conscious choice – at the outset – that is important. Theory helps guide research, can underpin methodology, and can help understand what is already known on the topic.^4^ There are two main types of theories, which although distinct, are complementary: explanatory and change theory. Explanatory theories (e.g., health belief model) seek to explain why a person acts or feels a certain way. Whilst important this information isn’t enough to promote a change in behaviour to improve that persons’ health which is why the application of a change theory is also necessary (e.g., transtheoretical model). Both types of theory – explanatory and change theories – can be used in qualitative research. Explanatory theory can guide the methodology, for example, guiding interview research questions to focus on the explanation of a particular phenomenon and/or providing the structure to a framework which can be used in thematic analysis of interview data. Change theory can also be applied in a similar way. For example, in designing a qualitative evaluation of a service to understand ‘what works’. Additionally, it can be used alongside other methods to qualitatively explain what is going on; for example, understanding the causal mechanisms that may be ‘in play’ in a randomised controlled trial.^5^

In their paper ‘The experience of caregiver burden when being next of kin to a person with severe chronic obstructive pulmonary disease: A Qualitative study’,^6^ the authors, Johansson et al., did not apply theory. Qualitative interviews were conducted with next of kin and narratives described the fluidity of caregiver roles whilst their own lives were put on hold. Observing the affected person struggle led to feelings of helplessness and frustration which were coupled with a sense of guilt and shame about viewing symptoms as disgusting. Perhaps Johansson et al. did not use theory because the aim was not to change a specific behaviour. However, the application of a change theory, alongside an explanatory theory, may have helped to guide a greater depth of understanding about the experience of caring for a person with COPD, what their needs are and what changes need to be made within the health system to enable these individuals to be better supported.

The authors are not alone in choosing not to apply theory to their qualitative study. Theories are seldom applied in research published within the field of respiratory medicine. This may be because as clinician scientists, health theories are often not included as part of training and as a result individuals do not feel they have the knowledge or skills to apply them, perhaps the value of applying theory in this way is not even fully understood. Or is it, as Crabtree and Millar argue, that clinicians view themselves to be working directly with reality; giving the example of “Blood is blood and diabetes is diabetes”,^7^ and instead prefer to draw on extensive clinical experience. Whilst research, even qualitative research, understands a biomedical model of the world, there are some ‘things’ which are objective in nature (such as blood) and the application of theory to qualitative research helps to generate a deeper level of understanding.^4^ The central role of theory in this manner is nicely discussed by Collins and Stockton (2018)^8^ who assert that whilst you may not think you are applying a theory, you almost certainly are, just by the way that you view the world. Therefore, having a conscious consideration of theory would seem pertinent and necessary to avoid bias and unintended impacts on the research process.

With this in mind, the study by Johansson et al. could have applied a theoretical lens/underpinning to elicit a deeper understanding of caregiver burden. One example could be the application of the COM-B model, which looks at capability (e.g., acquiring the skills to effectively care for next of kin), opportunity (e.g., having sufficient time to devote to care), and motivation (e.g., emotional burden of providing care) as an adjunct to overall behaviour (caring for next of kin with COPD), to understand the context and bring about change.^9^An example from respiratory medicine that has used the COM-B model effectively - ‘Facilitators and barriers to clinicians’ use of COPD action plans in self-management support: A qualitative study’ by Feiring and Friis (2019)^10^ - illustrates that the use of this theoretical model was applied to better understand clinicians’ views, which led to an analytical approach and findings which – because they systematically applied the theory – could be better compared to results of other studies, helping to pool and strengthen an evidence base.

To conclude, theory utilisation can be helpful. It doesn’t always have to be used, but it is worth considering. Ultimately, theories are useful for health care, health policy, and the care of patients and their carers. They help guide research so that clinicians can have a deeper and broader understanding of a clinical situation/phenomena. Theory can also help understand individual-level care, but with the possibility of applying it at a much broader, population-level.^2^ What is lost by not using theory?

## References

[1] Business Research Methodology . Theory. Available from: https://research-methodology.net/theory/ ((nd), accessed 3 May 2023).

[2] Reeves S Albert M Kuper A , et al. Why use theories in qualitative research? BMJ 2008; 337: a949. DOI: 10.1136/bmj.a949 18687730

[3] Stewart D Klein S . The use of theory in research. Int J Clin Pharm 2016; 38(3): 615–619. PMID: 26511946. DOI: 10.1007/s11096-015-0216-y 26511946

[4] Kelly M . The role of theory in qualitative health research. Fam Pract 2010; 27(3): 285–290. DOI: 10.1093/fampra/cmp077 19875746

[5] Todd L (ed). Theory-based methodology: using theories of change in educational development, research and evaluation. Newcastle upon Tyne, UK: Research Centre for Learning and Teaching, Newcastle University, 2015.

[6] Johansson H Berterö C Jonasson LL , et al. The experience of caregiver burden when being next of kin to a person with severe chronic obstructive pulmonary disease: a qualitative study. Chron Respir Dis 2023; 20: 14799731231168897. PMID: 37042067; PMCID: PMC10107968. DOI: 10.1177/14799731231168897 37042067 PMC10107968

[7] Crabtree BF Miller WL . Introduction. Doing qualitative research. Thousand Oaks, CA: Sage, 1999, pp. xi–xvii.

[8] Collins CS Stockton CM . The central role of theory in qualitative research. Int J Qual Methods 2018; 17: 160940691879747.

[9] Feiring E Friis T . Facilitators and barriers to clinicians’ use of COPD action plans in self-management support: a qualitative study. Patient Educ Couns 2020; 103(4): 693–701. Epub 2019 Nov 6. PMID: 31733986. DOI: 10.1016/j.pec.2019.11.002 31733986

[10] Michie S van Stralen MM West R . The behaviour change wheel: a new method for characterising and designing behaviour change interventions. Implement Sci 2011; 6: 42. DOI: 10.1186/1748-5908-6-42 21513547 PMC3096582

